# 2,2,2-Tribromo-*N*-(4-methyl­phen­yl)acetamide

**DOI:** 10.1107/S1600536810009177

**Published:** 2010-03-17

**Authors:** B. Thimme Gowda, Sabine Foro, P. A. Suchetan, Hartmut Fuess

**Affiliations:** a Department of Chemistry, Mangalore University, Mangalagangotri 574 199, Mangalore, India; b Institute of Materials Science, Darmstadt University of Technology, Petersenstrasse 23, D-64287 Darmstadt, Germany

## Abstract

The asymmetric unit of the title compound, C_9_H_8_Br_3_NO, contains two independent mol­ecules which differ in the orientation of the tribromo group. A weak intra­molecular N—H⋯Br hydrogen bond is observed in each mol­ecule. In the crystal, the independent mol­ecules are linked into chains along the *b* axis by inter­molecular N—H⋯O hydrogen bonds.

## Related literature

For the preparation of the title compound, see: Gowda *et al.* (2003 ▶). For our study of the effect of ring and the side-chain substituents on the solid-state structures of *N*-aromatic amides and for similar structures, see: Brown (1966 ▶); Gowda *et al.* (2009*a* ▶,*b* ▶,*c* ▶).
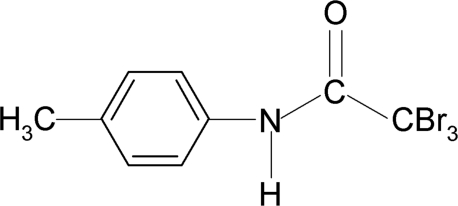


## Experimental

### 

#### Crystal data

C_9_H_8_Br_3_NO
*M* *_r_* = 385.89
Monoclinic, 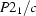

*a* = 9.6926 (6) Å
*b* = 20.531 (1) Å
*c* = 11.8139 (8) Å
β = 102.664 (7)°
*V* = 2293.8 (2) Å^3^
*Z* = 8
Mo *K*α radiation
μ = 10.52 mm^−1^
*T* = 299 K
0.48 × 0.40 × 0.30 mm

#### Data collection

Oxford Diffraction Xcalibur diffractometer with a Sapphire CCD detector
Absorption correction: multi-scan (*CrysAlis RED*; Oxford Diffraction, 2009 ▶) *T*_min_ = 0.081, *T*_max_ = 0.145
14714 measured reflections
4121 independent reflections
3375 reflections with *I* > 2σ(*I*)
*R*_int_ = 0.089

#### Refinement

*R*[*F* ^2^ > 2σ(*F* ^2^)] = 0.082
*wR*(*F* ^2^) = 0.151
*S* = 1.28
4121 reflections
255 parameters
H-atom parameters constrained
Δρ_max_ = 1.34 e Å^−3^
Δρ_min_ = −0.88 e Å^−3^

### 

Data collection: *CrysAlis CCD* (Oxford Diffraction, 2009 ▶); cell refinement: *CrysAlis RED* (Oxford Diffraction, 2009 ▶); data reduction: *CrysAlis RED*; program(s) used to solve structure: *SHELXS97* (Sheldrick, 2008 ▶); program(s) used to refine structure: *SHELXL97* (Sheldrick, 2008 ▶); molecular graphics: *PLATON* (Spek, 2009 ▶); software used to prepare material for publication: *SHELXL97*.

## Supplementary Material

Crystal structure: contains datablocks I, global. DOI: 10.1107/S1600536810009177/ci5053sup1.cif

Structure factors: contains datablocks I. DOI: 10.1107/S1600536810009177/ci5053Isup2.hkl

Additional supplementary materials:  crystallographic information; 3D view; checkCIF report

## Figures and Tables

**Table 1:** Hydrogen-bond geometry (Å, °)

*D*—H⋯*A*	*D*—H	H⋯*A*	*D*⋯*A*	*D*—H⋯*A*
N1—H1*N*⋯O2	0.86	2.27	3.078 (10)	156
N1—H1*N*⋯Br2	0.86	2.61	3.111 (8)	118
N2—H2*N*⋯O1^i^	0.86	2.27	3.032 (10)	148
N2—H2*N*⋯Br4	0.86	2.56	3.051 (9)	118

Symmetry code: (i) 
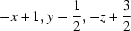
.

**Fig. 1.**
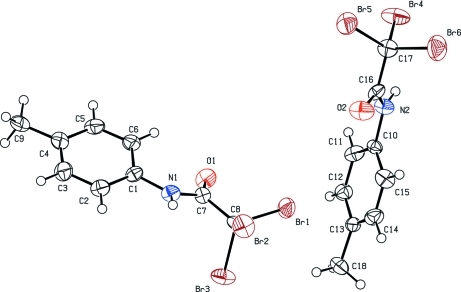
The asymmetric unit of the title compound, showing the atom labelling scheme. Displacement ellipsoids are drawn at the 50% probability level and H atoms are represented as small spheres of arbitrary radii.

**Fig. 2.**
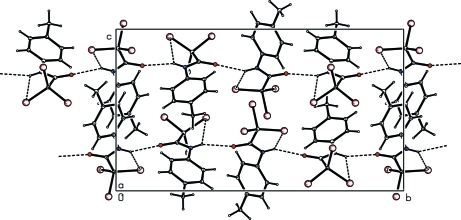
Molecular packing in the crystal structure of the title compound, with hydrogen bonds shown as dashed lines.

## supplementary crystallographic information

### Comment

As part of a study of the effect of the ring and the side chain substituents on solid state structures of *N*-aromatic amides (Gowda *et al.*, 2009*a*,*b*,*c*), in the present work, the crytsal structure of 2,2,2-tribromo-*N*-(4-methylphenyl)acetamide has been determined (Fig.1). The asymmetric unit of the structure contains two independent molecules, which differ in the orientation of the tribromo group as is evident from either the C-N-CO-CBr3 or N-CO-C-Br torsional angles. The conformations of the N—H bonds in both molecules are *anti* to the C═O bonds in the side chains, similar to those observed in 2,2,2-tribromo-*N*-(3-methylphenyl)acetamide (Gowda *et al.*, 2009*a*), 2,2,2-tribromo-*N*-(phenyl)acetamide (Gowda *et al.*, 2009*b*), 2,2,2-tribromo-*N*-(4-chlorophenyl)acetamide (Gowda *et al.*, 2009*c*) and other amides (Brown, 1966). The structure of the title compound shows both the intramolecular N—H···Br and intermolecular N—H···O hydrogen bonding.

The packing diagram of molecules showing the hydrogen bonds N1—H1N···O2 and N2—H2N···O1 (Table 1) involved in the formation of molecular chains is shown in Fig. 2.

### Experimental

The title compound was prepared from *p*-toluidine, tribromoacetic acid and phosphorylchloride according to the literature method (Gowda *et al.*, 2003). The purity of the compound was checked by determining its melting point. It was further characterized by recording its infrared spectra. Single crystals of the title compound used for X-ray diffraction studies were obtained by a slow evaporation of its solution in petroleum ether at room temperature.

### Refinement

H atoms were positioned with idealized geometry using a riding model with C–H = 0.93–0.96 Å, N–H = 0.86 Å and U_iso_(H) = 1.2U_eq_(parent atom). The residual electron-density features are located in the region of Br4 and Br3. The highest peak is 0.99 Å from Br4 and the deepest hole is 1.28 Å from Br3. Owing to the poor diffraction quality of the crystal, the R_int_ value is high (0.089) and this is a structure of relatively low precision.

### Crystal data

**Table:**

C_9_H_8_Br_3_NO	*F*(000) = 1456
*M**_r_* = 385.89	*D*_x_ = 2.235 Mg m^−^^3^
Monoclinic, *P*2_1_/*c*	Mo *K*α radiation, λ = 0.71073 Å
Hall symbol: -P 2ybc	Cell parameters from 5162 reflections
*a* = 9.6926 (6) Å	θ = 2.7–27.8°
*b* = 20.531 (1) Å	µ = 10.52 mm^−^^1^
*c* = 11.8139 (8) Å	*T* = 299 K
β = 102.664 (7)°	Prism, colourless
*V* = 2293.8 (2) Å^3^	0.48 × 0.40 × 0.30 mm
*Z* = 8	

### Data collection

**Table:**

Oxford Diffraction Xcalibur diffractometer with a Sapphire CCD detector	4121 independent reflections
Radiation source: fine-focus sealed tube	3375 reflections with *I* > 2σ(*I*)
graphite	*R*_int_ = 0.089
Rotation method data acquisition using ω and φ scans.	θ_max_ = 25.4°, θ_min_ = 4.2°
Absorption correction: multi-scan (*CrysAlis RED*; Oxford Diffraction, 2009)	*h* = −11→11
*T*_min_ = 0.081, *T*_max_ = 0.145	*k* = −21→24
14714 measured reflections	*l* = −13→14

### Refinement

**Table:**

Refinement on *F*^2^	Primary atom site location: structure-invariant direct methods
Least-squares matrix: full	Secondary atom site location: difference Fourier map
*R*[*F*^2^ > 2σ(*F*^2^)] = 0.082	Hydrogen site location: inferred from neighbouring sites
*wR*(*F*^2^) = 0.151	H-atom parameters constrained
*S* = 1.28	*w* = 1/[σ^2^(*F*_o_^2^) + (0.*P*)^2^ + 26.8682*P*] where *P* = (*F*_o_^2^ + 2*F*_c_^2^)/3
4121 reflections	(Δ/σ)_max_ = 0.001
255 parameters	Δρ_max_ = 1.34 e Å^−^^3^
0 restraints	Δρ_min_ = −0.88 e Å^−^^3^

### Special details

**Table:**

Geometry. All esds (except the esd in the dihedral angle between two l.s. planes) are estimated using the full covariance matrix. The cell esds are taken into account individually in the estimation of esds in distances, angles and torsion angles; correlations between esds in cell parameters are only used when they are defined by crystal symmetry. An approximate (isotropic) treatment of cell esds is used for estimating esds involving l.s. planes.
Refinement. Refinement of *F*^2^ against ALL reflections. The weighted *R*-factor wR and goodness of fit *S* are based on *F*^2^, conventional *R*-factors *R* are based on *F*, with *F* set to zero for negative *F*^2^. The threshold expression of *F*^2^ > σ(*F*^2^) is used only for calculating *R*-factors(gt) etc. and is not relevant to the choice of reflections for refinement. *R*-factors based on *F*^2^ are statistically about twice as large as those based on *F*, and *R*- factors based on ALL data will be even larger.

### Fractional atomic coordinates and isotropic or equivalent isotropic displacement parameters (Å^2^)

**Table:**

	*x*	*y*	*z*	*U*_iso_*/*U*_eq_	
C1	0.7165 (11)	0.2486 (4)	0.6954 (9)	0.029 (2)	
C2	0.7414 (11)	0.1962 (5)	0.6292 (10)	0.035 (3)	
H2	0.6948	0.1569	0.6335	0.043*	
C3	0.8348 (12)	0.2017 (5)	0.5568 (11)	0.043 (3)	
H3	0.8516	0.1658	0.5136	0.051*	
C4	0.9039 (11)	0.2598 (5)	0.5475 (9)	0.034 (2)	
C5	0.8797 (12)	0.3110 (5)	0.6175 (10)	0.039 (3)	
H5	0.9279	0.3500	0.6149	0.047*	
C6	0.7875 (12)	0.3065 (5)	0.6901 (10)	0.037 (3)	
H6	0.7730	0.3419	0.7353	0.044*	
C7	0.5389 (11)	0.2876 (4)	0.7992 (9)	0.028 (2)	
C8	0.4213 (11)	0.2643 (4)	0.8592 (9)	0.029 (2)	
C9	1.0009 (12)	0.2673 (6)	0.4652 (10)	0.043 (3)	
H9A	0.9464	0.2667	0.3869	0.052*	
H9B	1.0675	0.2320	0.4762	0.052*	
H9C	1.0507	0.3079	0.4800	0.052*	
Br1	0.37301 (14)	0.33225 (5)	0.95617 (12)	0.0480 (4)	
Br2	0.46539 (15)	0.18686 (5)	0.95318 (12)	0.0510 (4)	
Br3	0.25450 (14)	0.24690 (6)	0.73598 (12)	0.0531 (4)	
N1	0.6157 (9)	0.2404 (4)	0.7652 (8)	0.033 (2)	
H1N	0.6029	0.2015	0.7877	0.039*	
O1	0.5509 (8)	0.3449 (3)	0.7822 (7)	0.042 (2)	
C10	0.3798 (10)	0.0087 (4)	0.6530 (9)	0.025 (2)	
C11	0.3488 (11)	−0.0253 (5)	0.5503 (10)	0.036 (3)	
H11	0.4045	−0.0606	0.5390	0.044*	
C12	0.2340 (11)	−0.0068 (5)	0.4633 (10)	0.034 (3)	
H12	0.2144	−0.0297	0.3937	0.041*	
C13	0.1478 (10)	0.0452 (5)	0.4785 (9)	0.029 (2)	
C14	0.1792 (12)	0.0765 (5)	0.5829 (11)	0.038 (3)	
H14	0.1220	0.1108	0.5960	0.046*	
C15	0.2935 (12)	0.0590 (5)	0.6701 (10)	0.035 (3)	
H15	0.3117	0.0813	0.7403	0.042*	
C16	0.5967 (10)	0.0331 (5)	0.7916 (10)	0.030 (2)	
C17	0.7240 (12)	0.0074 (5)	0.8818 (10)	0.036 (3)	
C18	0.0294 (13)	0.0685 (5)	0.3837 (10)	0.042 (3)	
H18A	0.0032	0.0346	0.3270	0.050*	
H18B	0.0595	0.1062	0.3475	0.050*	
H18C	−0.0504	0.0795	0.4157	0.050*	
Br4	0.75931 (14)	−0.08465 (6)	0.87190 (14)	0.0582 (4)	
Br5	0.89309 (14)	0.05436 (7)	0.86845 (12)	0.0540 (4)	
Br6	0.69036 (17)	0.02579 (8)	1.03563 (12)	0.0661 (4)	
N2	0.5023 (9)	−0.0097 (4)	0.7398 (8)	0.032 (2)	
H2N	0.5144	−0.0501	0.7586	0.038*	
O2	0.5901 (9)	0.0912 (3)	0.7746 (8)	0.049 (2)	

### Atomic displacement parameters (Å^2^)

**Table:**

	*U*^11^	*U*^22^	*U*^33^	*U*^12^	*U*^13^	*U*^23^
C1	0.024 (5)	0.027 (5)	0.037 (7)	0.003 (4)	0.006 (5)	0.004 (4)
C2	0.032 (6)	0.025 (5)	0.049 (8)	0.002 (4)	0.007 (6)	−0.001 (5)
C3	0.039 (7)	0.034 (6)	0.057 (9)	0.005 (5)	0.015 (6)	−0.011 (5)
C4	0.031 (6)	0.045 (6)	0.020 (6)	0.008 (5)	−0.004 (5)	0.007 (5)
C5	0.032 (6)	0.036 (6)	0.049 (8)	−0.008 (5)	0.006 (6)	−0.003 (5)
C6	0.035 (6)	0.033 (5)	0.043 (7)	−0.006 (5)	0.008 (6)	−0.009 (5)
C7	0.030 (6)	0.027 (5)	0.028 (6)	0.001 (4)	0.006 (5)	0.001 (4)
C8	0.030 (6)	0.022 (5)	0.029 (6)	0.005 (4)	−0.008 (5)	−0.002 (4)
C9	0.035 (7)	0.055 (7)	0.035 (7)	−0.003 (6)	−0.004 (6)	−0.009 (6)
Br1	0.0487 (7)	0.0402 (6)	0.0610 (9)	−0.0018 (5)	0.0251 (7)	−0.0155 (6)
Br2	0.0660 (9)	0.0386 (6)	0.0484 (8)	0.0034 (6)	0.0128 (7)	0.0101 (6)
Br3	0.0423 (7)	0.0586 (8)	0.0496 (9)	−0.0124 (6)	−0.0086 (6)	−0.0035 (6)
N1	0.035 (5)	0.015 (4)	0.049 (6)	0.000 (4)	0.009 (5)	−0.002 (4)
O1	0.042 (5)	0.021 (4)	0.064 (6)	0.004 (3)	0.017 (4)	0.001 (3)
C10	0.024 (5)	0.022 (5)	0.027 (6)	−0.003 (4)	0.001 (5)	0.004 (4)
C11	0.027 (6)	0.030 (5)	0.051 (8)	0.001 (5)	0.007 (6)	−0.002 (5)
C12	0.032 (6)	0.037 (6)	0.032 (7)	0.000 (5)	0.004 (5)	−0.003 (5)
C13	0.024 (5)	0.028 (5)	0.034 (7)	0.000 (4)	0.007 (5)	0.008 (5)
C14	0.035 (6)	0.028 (5)	0.053 (8)	0.007 (5)	0.014 (6)	0.003 (5)
C15	0.048 (7)	0.026 (5)	0.030 (7)	−0.001 (5)	0.002 (6)	−0.010 (5)
C16	0.023 (5)	0.026 (5)	0.044 (7)	−0.001 (4)	0.012 (5)	−0.003 (5)
C17	0.034 (6)	0.026 (5)	0.044 (7)	−0.009 (5)	0.001 (6)	−0.004 (5)
C18	0.045 (7)	0.046 (6)	0.031 (7)	0.006 (6)	0.002 (6)	0.009 (5)
Br4	0.0472 (8)	0.0352 (6)	0.0788 (11)	0.0055 (5)	−0.0149 (7)	0.0032 (6)
Br5	0.0393 (7)	0.0677 (8)	0.0523 (9)	−0.0199 (6)	0.0043 (6)	−0.0008 (7)
Br6	0.0644 (10)	0.0966 (11)	0.0391 (8)	−0.0102 (8)	0.0150 (7)	−0.0027 (7)
N2	0.033 (5)	0.018 (4)	0.040 (6)	−0.002 (4)	−0.001 (4)	−0.001 (4)
O2	0.044 (5)	0.022 (4)	0.076 (7)	−0.008 (3)	−0.001 (5)	0.005 (4)

### Geometric parameters (Å, °)

**Table:**

C1—C2	1.383 (14)	C10—C15	1.371 (13)
C1—C6	1.382 (13)	C10—C11	1.374 (14)
C1—N1	1.420 (12)	C10—N2	1.439 (13)
C2—C3	1.380 (15)	C11—C12	1.392 (15)
C2—H2	0.93	C11—H11	0.93
C3—C4	1.384 (15)	C12—C13	1.392 (13)
C3—H3	0.93	C12—H12	0.93
C4—C5	1.389 (14)	C13—C14	1.366 (15)
C4—C9	1.501 (15)	C13—C18	1.495 (15)
C5—C6	1.371 (15)	C14—C15	1.384 (16)
C5—H5	0.93	C14—H14	0.93
C6—H6	0.93	C15—H15	0.93
C7—O1	1.201 (11)	C16—O2	1.209 (11)
C7—N1	1.337 (12)	C16—N2	1.318 (13)
C7—C8	1.545 (14)	C16—C17	1.536 (15)
C8—Br1	1.927 (9)	C17—Br4	1.928 (10)
C8—Br2	1.932 (9)	C17—Br5	1.938 (10)
C8—Br3	1.957 (10)	C17—Br6	1.953 (11)
C9—H9A	0.96	C18—H18A	0.96
C9—H9B	0.96	C18—H18B	0.96
C9—H9C	0.96	C18—H18C	0.96
N1—H1N	0.86	N2—H2N	0.86
			
C2—C1—C6	119.6 (9)	C15—C10—C11	119.3 (10)
C2—C1—N1	117.7 (8)	C15—C10—N2	121.8 (9)
C6—C1—N1	122.8 (9)	C11—C10—N2	118.8 (9)
C3—C2—C1	120.5 (9)	C10—C11—C12	119.9 (10)
C3—C2—H2	119.8	C10—C11—H11	120.1
C1—C2—H2	119.8	C12—C11—H11	120.1
C2—C3—C4	120.9 (10)	C13—C12—C11	121.3 (10)
C2—C3—H3	119.5	C13—C12—H12	119.4
C4—C3—H3	119.5	C11—C12—H12	119.4
C3—C4—C5	117.2 (10)	C14—C13—C12	117.2 (10)
C3—C4—C9	121.5 (10)	C14—C13—C18	120.6 (9)
C5—C4—C9	121.3 (10)	C12—C13—C18	122.2 (10)
C6—C5—C4	122.7 (10)	C13—C14—C15	122.2 (10)
C6—C5—H5	118.7	C13—C14—H14	118.9
C4—C5—H5	118.7	C15—C14—H14	118.9
C5—C6—C1	119.1 (10)	C10—C15—C14	120.1 (10)
C5—C6—H6	120.5	C10—C15—H15	120.0
C1—C6—H6	120.5	C14—C15—H15	120.0
O1—C7—N1	125.4 (9)	O2—C16—N2	125.0 (11)
O1—C7—C8	119.2 (8)	O2—C16—C17	117.4 (9)
N1—C7—C8	115.3 (8)	N2—C16—C17	117.6 (8)
C7—C8—Br1	110.4 (6)	C16—C17—Br4	114.9 (7)
C7—C8—Br2	115.2 (6)	C16—C17—Br5	109.7 (7)
Br1—C8—Br2	107.8 (5)	Br4—C17—Br5	108.5 (5)
C7—C8—Br3	106.7 (7)	C16—C17—Br6	107.9 (7)
Br1—C8—Br3	107.8 (5)	Br4—C17—Br6	108.3 (5)
Br2—C8—Br3	108.7 (5)	Br5—C17—Br6	107.2 (5)
C4—C9—H9A	109.5	C13—C18—H18A	109.5
C4—C9—H9B	109.5	C13—C18—H18B	109.5
H9A—C9—H9B	109.5	H18A—C18—H18B	109.5
C4—C9—H9C	109.5	C13—C18—H18C	109.5
H9A—C9—H9C	109.5	H18A—C18—H18C	109.5
H9B—C9—H9C	109.5	H18B—C18—H18C	109.5
C7—N1—C1	126.0 (8)	C16—N2—C10	122.4 (8)
C7—N1—H1N	117.0	C16—N2—H2N	118.8
C1—N1—H1N	117.0	C10—N2—H2N	118.8
			
C6—C1—C2—C3	−1.0 (17)	C15—C10—C11—C12	2.5 (14)
N1—C1—C2—C3	178.0 (10)	N2—C10—C11—C12	−177.6 (9)
C1—C2—C3—C4	−1.0 (18)	C10—C11—C12—C13	−0.8 (15)
C2—C3—C4—C5	2.7 (17)	C11—C12—C13—C14	−1.2 (14)
C2—C3—C4—C9	−177.3 (11)	C11—C12—C13—C18	176.1 (10)
C3—C4—C5—C6	−2.6 (17)	C12—C13—C14—C15	1.5 (15)
C9—C4—C5—C6	177.4 (11)	C18—C13—C14—C15	−175.8 (10)
C4—C5—C6—C1	0.7 (18)	C11—C10—C15—C14	−2.2 (15)
C2—C1—C6—C5	1.2 (17)	N2—C10—C15—C14	177.9 (9)
N1—C1—C6—C5	−177.8 (11)	C13—C14—C15—C10	0.1 (16)
O1—C7—C8—Br1	−26.3 (12)	O2—C16—C17—Br4	−162.1 (8)
N1—C7—C8—Br1	156.8 (8)	N2—C16—C17—Br4	18.7 (12)
O1—C7—C8—Br2	−148.7 (9)	O2—C16—C17—Br5	−39.5 (12)
N1—C7—C8—Br2	34.4 (12)	N2—C16—C17—Br5	141.3 (8)
O1—C7—C8—Br3	90.6 (10)	O2—C16—C17—Br6	77.0 (10)
N1—C7—C8—Br3	−86.3 (9)	N2—C16—C17—Br6	−102.2 (9)
O1—C7—N1—C1	−5.5 (18)	O2—C16—N2—C10	1.6 (16)
C8—C7—N1—C1	171.2 (10)	C17—C16—N2—C10	−179.3 (9)
C2—C1—N1—C7	−153.2 (11)	C15—C10—N2—C16	−49.1 (14)
C6—C1—N1—C7	25.8 (17)	C11—C10—N2—C16	130.9 (10)

### Hydrogen-bond geometry (Å, °)

**Table:**

*D*—H···*A*	*D*—H	H···*A*	*D*···*A*	*D*—H···*A*
N1—H1N···O2	0.86	2.27	3.078 (10)	156
N1—H1N···Br2	0.86	2.61	3.111 (8)	118
N2—H2N···O1^i^	0.86	2.27	3.032 (10)	148
N2—H2N···Br4	0.86	2.56	3.051 (9)	118

Symmetry codes: (i) −*x*+1, *y*−1/2, −*z*+3/2.
